# Analysis of Differences in User Groups and Post Sentiment of COVID-19 Vaccine Hesitators in Chinese Social-Media Platforms

**DOI:** 10.3390/healthcare11091207

**Published:** 2023-04-23

**Authors:** Jingfang Liu, Shuangjinhua Lu, Huiqin Zheng

**Affiliations:** School of Management, Shanghai University, No. 20, Chengzhong Road, Jiading District, Shanghai 201899, China

**Keywords:** user groups, sentiment analysis, vaccine hesitancy, COVID-19 vaccine

## Abstract

(1) Background: The COVID-19 epidemic is still global and no specific drug has been developed for COVID-19. Vaccination can both prevent infection and limit the spread of the epidemic. Eliminating hesitation to the COVID-19 vaccine and achieving early herd immunity is a common goal for all countries. However, efforts in this area have not been significant and there is still a long way to go to eliminate vaccine hesitancy. (2) Objective: This study aimed to uncover differences in the characteristics and sentiments of COVID-19 vaccine hesitators on Chinese social-media platforms and to achieve a classification of vaccine-hesitant groups. (3) Methods: COVID-19-vaccine-hesitation posts and user characteristics were collected on the Sina Microblog platform for posting times spanning one year, and posts were identified for hesitation types. Logistic regression was used to conduct user-group analysis. The differences in user characteristics between the various types of COVID-19 vaccine posts were analysed according to four user characteristics: gender, address type, degree of personal-information disclosure, and whether they followed health topics. Sentiment analysis was conducted using sentiment analysis tools to calculate the sentiment scores and sentiment polarity of various COVID-19 vaccine posts, and the K–W test was used to uncover the sentiment differences between various types of COVID-19-vaccine-hesitation posts. (4) Results: There are differences in the types of COVID-19-vaccine-hesitation posts posted by users with different characteristics, and different types of COVID-19-vaccine-hesitation posts differ in terms of sentiment. Differences in user attributes and user behaviors are found across the different COVID-19-vaccine-hesitation types. Ultimately, two COVID-19-vaccine-hesitant user groups were identified: Body-related and Non-bodily-related. Users who posted body-related vaccine-hesitation posts are more often female, disclose more personal information and follow health topics on social-media platforms. Users who posted non-bodily-related posts are more often male, disclose less personal information, and do not follow health topics. The average sentiment score for all COVID-19-vaccine-hesitant-type posts is less than 0.45, with negative-sentiment posts outweighing positive- and neutral-sentiment posts in each type, among which the “Individual rights” type is the most negative. (5) Conclusions: This paper complements the application of user groups in the field of vaccine hesitation, and the results of the analysis of group characteristics and post sentiment can help to provide an in-depth and comprehensive analysis of the concerns and needs of COVID-19 vaccine hesitators. This will help public-health agencies to implement more targeted strategies to eliminate vaccine hesitancy and improve their work related to the COVID-19 vaccine, with far-reaching implications for COVID-19-vaccine promotion and vaccination.

## 1. Introduction

The COVID-19 epidemic, which spread rapidly worldwide and continues to this day, has become a serious threat to human health and has had a major impact on social and economic development. As of 7 January 2023, the COVID-19 pandemic has included more than 657 million confirmed cases and has caused more than 6.68 million deaths worldwide [1,2].

COVID-19 is an infectious disease caused by coronavirus-2 (SARS-CoV-2), which is highly infectious and has a low cure rate [3,4]. The course of SARS-CoV-2 infection is relatively insidious and the manifestation of symptoms after infection can range from mild to severe. Those with mild infection can recover in about two weeks with only mild upper respiratory symptoms or no symptomatic manifestations, while those with severe infection have a recovery time of about three to six weeks and may develop pneumonia and acute respiratory distress syndrome (ARDS), or even die [3,5,6]. Even after recovery, it has been shown that COVID-19 causes long-term damage to multiple organs [7], with over 70% of survivors experiencing long-term persistent symptoms and potential sequelae, known as post-COVID syndrome [8], with common symptoms such as fatigue, cough, chest tightness, dyspnea, cognitive impairment, anxiety and depression [7,8,9]. Post-COVID syndrome reduces the quality of life of patients and it is not clear which patients are most likely to develop post-COVID syndrome and whether these symptoms will be ameliorated or become a permanent problem [8]. Currently, no specific drug has been developed for COVID-19 [5,10,11] and treatment has mostly focused on supportive care, symptomatic treatment and therapeutic and clinical trials with drugs already on the market, and boosting the immunity of patients [4,6]. As there is no curative treatment, prevention is, thus, the main way to interrupt the spread of the SARS-CoV-2 and limit the spread of the epidemic [12]. Vaccination has proven to be one of the most outstanding achievements in the field of public health [13], and in the current global fight against the COVID-19 pandemic, the COVID-19 vaccine has become the most effective weapon for prevention [14].

Safe and effective vaccines provide excellent prevention of serious illness, hospitalisation and death caused by COVID-19, and the efficacy of most vaccines against severe illness remains at a high level of more than 70% for an extended period when fully vaccinated [15,16]. Viral mutations are inevitable over time and as the number of infections increases [17,18], and studies have shown that complete vaccination and vaccination boosting are still effective in preventing infection with mutant strains of SARS-CoV-2 and help reduce the likelihood of new viral-lineage evolution [16,19]. Positive patients who were vaccinated have a shorter healing time and lower viral load, which, to some extent, reduces the transmission of the virus and alleviates the limited medical resources available, which is significant in the current and future pandemic context [17,20].

Therefore, vaccination implementation is critical. The World Health Organization actively promotes increased COVID-19-vaccine accessibility, helps countries to accelerate vaccine delivery, and advocates that countries reach at least 70% vaccination coverage [21] (The vaccination coverage threshold for achieving herd immunity needs to be between 55% and 82% of the total population factors [22]). Countries around the world have responded positively to the call for large-scale vaccination campaigns, but the current vaccination situation is not promising, with COVID-19 vaccination rates varying widely across the globe, with most countries still below target levels, and almost all countries yet to establish herd immunity [23,24]. Due to the safety and efficacy of the COVID-19 vaccine [25], religious beliefs [26], conspiracy theories [27], and other reasons, not everyone is willing to be vaccinated, that is, there is vaccine hesitation. In China, the main reasons affecting the hesitation to vaccinate against COVID-19 are not only the safety, efficacy, and necessity of the vaccine [28], the presence of elderly and children in families [29,30], the guidance of government policies and the level of trust in the government [31,32], whether the job position faces a greater risk of infection [32], and the physical health status such as the presence of underlying diseases [33,34] but also other reasons which can also influence people’s willingness to get vaccinated. The SAGE Working Group reported vaccine hesitancy as the choice to delay or refuse vaccination despite the availability of vaccination services [35]. Vaccine hesitation may be a major barrier to COVID-19 vaccination and the establishment of herd immunity. The causes of vaccine hesitancy are complex and homeopathic, influenced by time, place, vaccine type, misinformation, convenience, trust [36], and a single intervention cannot cope with all causes of vaccine hesitancy; specific analysis is needed to uncover the characteristics and differences in COVID-19 vaccine hesitators, achieve segmentation of vaccine hesitator groups, explore individual characteristics of vaccine hesitators and behavioral characteristics, and develop precise strategies to eliminate vaccine hesitancy and improve vaccination rates.

Social-media platforms have a unique role in public-crisis management and have become an important medium for information dissemination during the COVID-19 pandemic and have greatly influenced public attitudes towards vaccination [37]. Social-media platforms have a wealth of information reflecting public behavior and attitudes. By analyzing this data, it is possible to identify the behavioral characteristics and needs of citizens, thus providing timely and accurate information to the public and reducing public anxiety and fear [38]. This paper conducts user-group analysis of COVID-19 vaccine hesitators on social-media platforms and sentiment analysis of their posts on COVID-19 vaccine hesitation, to discover the differences in user characteristics behind the various types of hesitation-reason posts and the sentiment differences in the posts. The results on group characteristics and sentiment differences can help public-health agencies develop more targeted vaccine-hesitancy elimination programs.

## 2. Literature Review

### 2.1. Vaccine Hesitancy

The definition of vaccine hesitancy is still unclear in the academic field, and there seems to be a discrepancy between what is referred to as vaccine hesitancy in relevant articles. For example, the World Health Organization’s Advisory Group of Experts on Immunization Strategies advocated a continuum of public attitudes toward vaccination, with the vaccine-hesitant group being a continuum between “complete refusal” and “complete willingness” [35]. Some scholars defined vaccine hesitancy as the choice to delay or refuse vaccination despite its accessibility [39]. Peretti-Watel et al. argued that vaccine hesitancy is an ambiguous concept that they consider as a decision-making process and pointed out that the definition of vaccine hesitancy is often very broad and includes different people, different situations, and many different explanatory factors [40]. The philosopher Goldenberg asserted that vaccine hesitancy is an ambivalent attitude and that this ambivalence reflects the individual’s anxiety about the time and the culture of the region in which they live [41].

In this paper, we used posts related to COVID-19 vaccine hesitancy on social-media platforms. As the definition of vaccine hesitancy is ambiguous, we considered all posts that expressed hesitation, concern, doubt, or rejection about the vaccine as vaccine-hesitancy texts. On the one hand, it is difficult to strictly distinguish the semantics of hesitation and rejection in texts, it is difficult to develop classification criteria for hesitation and firm rejection, and we cannot verify whether posters have received the COVID-19 vaccine; on the other hand, Razai argued that although most vaccine hesitators on social media are not staunch anti-vaccine advocates or conspiracy theorists, they simply have not yet decided to get vaccinated for a specific reason [42], posters who do refuse vaccination may also change their minds.

Numerous studies have found that various demographic characteristics such as race, gender, age, and economic status are significantly associated with vaccine hesitancy. Hooper et al. found that the level of vaccine hesitancy varied between races, with African American and Latino adults having increased levels of vaccine hesitancy during the COVID-19 pandemic [43]. Robertson et al. found that Black people or Black British and Pakistani or Bangladeshi ethnic groups were the most hesitant [44]; a study by Latkin et al. reached similar conclusions: African-American and Hispanic respondents had lower vaccination intentions, in contrast to higher levels of vaccine hesitancy among women, young adults, and political conservatives [45]. A study that investigated COVID-19 vaccination intentions among groups with different demographic characteristics in Japan found that men showed less hesitation to vaccination [46]. Gunes assessed the attitudes of Turkish parents toward childhood vaccines and showed that the higher the level of education, the more negative news they heard about vaccines, thus leading to vaccine hesitation [47]. Lin et al. also agreed that higher levels of education are associated with higher levels of vaccine hesitation because they may be more skeptical of the COVID-19 vaccine [25]. However, Li et al. argued that highly educated individuals are usually capable of making the right vaccination decisions and, thus, less educated individuals are more hesitant about vaccines, but both scholars agreed that knowledge is an important factor influencing vaccine hesitancy, and Li also found that vaccine hesitation was influenced by age, with older individuals being more hesitant about vaccines [18]. Reiter et al. found that individuals with lower socioeconomic status or no health insurance were more likely to be vaccine-hesitant [48] and that aggressive vaccine payment policies may promote COVID-19 vaccination [49]. Low income is a barrier to vaccination, but it has also been shown that households with higher income levels are more able to cope with the adverse effects of disease and, thus, have higher levels of vaccine hesitancy [50]. We found differences in the findings of national scholars regarding the demographics of COVID-19 vaccine hesitancy, which shows that the causes of vaccine hesitancy are indeed complex.

### 2.2. User Groups

User groups appear most frequently in studies on the user persona. The concept of user persona was introduced by Cooper, the “father of interaction design”, and applied to the development and design of software [51]. It is essentially a group of similar users brought together to represent the goals, needs, and behaviors of that type of user [52], and is a virtual representation of a real user group [53,54]. As personas focus on the target user group and the right personas provide ideas and information that are better suited to the target user group, personas are often used as a tool to conceptualize user-centered design [55]. The process of developing personas can usually be divided into four stages: (1) identifying target users, (2) collecting user data, (3) grouping users, and (4) creating and presenting personas [56], of which step three is the most important. The results of user grouping are crucial to the validity of the final personas and need to reflect significant differences in the user groups [57].

User groups are mostly used in research related to improving user experience and product services. A customer segmentation method was developed by Jisun et al. By mining customer consumption-behavior data and demographic data online to identify customer groups for various business purposes, representative personas can be generated automatically to understand customer needs and preferences [58]. Tan et al. used online social platforms to extract user behavioral characteristics and relevant content of the target product for classification to form user groups, each with distinctly different characteristics. Six personas representing different user types were also constructed based on the user groups, thus supporting the design and optimization of the product [55]. Malakhatka et al. used semi-structured interviews and focus groups to identify user characteristics in online-shopping communities and to form initial user groupings. Finally four personas were developed by differentiating the characteristics of users based on their motivation to participate, reading behaviour and posting behaviour [56]. Almeshari et al. investigated the preferences and needs of different visitors to a museum through a questionnaire, distinguishing six groups of users representing different user types through two distinctive characteristics: motivation for visiting, and success criteria for visiting. Differences in characteristic preferences between different user types can help optimize museum visit guides and provide a better personalized user experience [59]. Yoo et al. found that meaningful user groups could be formed using the distribution characteristics of listeners’ play rates, and used two clustering algorithms to eventually form four user groups to respond to users’ listening behavior. This study provides new ideas for identifying and matching user types to help apply the right recommendation algorithm to provide them with a better personalized music-listening experience [60].

User groups have also been extended to include health-related research. Klooster et al. developed a semi-automatic user-grouping algorithm. The user groups were updated using the patient’s electronic medical-record data through a three-step clustering iteration, and representatives of each group were identified from the final user grouping to form patient personas [61]. To gain insight into the behavior and needs of the ASCVD population in Singapore and, thus, provide patient-centered healthcare interventions, Haldane et al. formed patient groups based on data on user characteristics such as demographics, socio-economic factors, social support, medication adherence, health literacy, information seeking and mobile-phone use. Five role types were also abstracted to represent the ASCVD patient population in Singapore [62]. Haupt et al. generated a model of seven groups through cluster analysis that represented individual differences in how US citizens perceived risk and adhered to social-distance guidelines during the COVID-19 pandemic. Differences in risk-taking propensities based on each group ultimately presented seven persona types that helped to individualize communication and policy guidance based on each persona’s demographic characteristics, psychological tendencies, and situational context [63]. Using a group of parents of adolescents aged nine to fourteen years in the United States, Massey et al. analyzed data sources and identified four potential user groups, ultimately abstracting four personas to represent types of parents with different attitudes regarding the HPV vaccine and decision-making. The aim was to use these personas to provide narrative health education on social media, thereby increasing parents’ willingness to vaccinate their children against HPV [64].

There has been relatively little research on the application of user groups to the vaccine scenario, especially concerning COVID-19 vaccine hesitancy. Hence, this study conducted a user-group analysis of COVID-19 vaccine hesitators on social platforms to identify differences in user characteristics behind different hesitation-reason texts, and the final results of the user groups can be further applied to COVID-19-vaccine-hesitant persona development.

### 2.3. Sentiment Analysis

Sentiment analysis aims to automatically mine a text for opinions, attitudes, and feelings, which is a powerful tool for monitoring important events and trends in real time [65]. Sentiment analysis has been used more frequently in social media texts in recent years than in online product reviews in the past [66]. There has been an explosion of discussion about COVID-19 on social media, and some research has suggested the critical importance of social media in the public’s vaccination decision-making process [67].

Many studies have used social-media sentiment analysis to monitor public attitudes and changes regarding COVID-19 vaccine-related issues in real-time. Based on text data from social-media platforms, Gao et al. used sentiment analysis to find that the public’s positive and negative sentiments increased simultaneously after the COVID-19 vaccine was approved for marketing, focusing on vaccine performance issues before official approval but more on vaccination issues afterward [68]. Ding et al. found that the intensity of public sentiment regarding the COVID-19 vaccine varied over time, with overall positive and negative sentiment being roughly equal in intensity, but there were differences in sentiment intensity when trending topics emerged [69]. Ansari et al. investigated geographic differences in sentiment regarding COVID-19 vaccination using relevant tweets by cutting into the analysis from demographic characteristics, finding that tweets were generally negative and that there was a huge global trend towards vaccination [70].

Scholars have also used a combination of topic analysis and sentiment analysis to identify trending topics and sentiment on social media related to the COVID-19 vaccine to better capture trends in the COVID-19 pandemic and understand public perceptions, concerns, and sentiment. Jang et al. applied a weakly supervised aspect-based sentiment-analysis technique to tweets related to the COVID-19 vaccination to investigate public attitudinal changes toward vaccination since the beginning of the vaccine rollout, determining the sentiment polarity of 20 key aspects, with the greatest negative sentiment being in response to adverse reactions to the vaccine [71]. Monselise et al. performed unsupervised sentiment analysis on the 12 topics with the most attention on social media regarding the COVID-19 vaccine, identifying positive and negative sentiment to determine the overall public reaction to the vaccine and news events related to the vaccine, and found that fear was the predominant emotion [72].

Some scholars have found a relationship between emotions and vaccination rates through sentiment analysis, and Yousef et al. found that the higher the incidence of negative emotions in public vaccination, the higher the vaccination rate [73]. Chien et al. found that trends in public vaccination emotions were significantly associated with increasing trends in vaccination rates and falling trends in COVID-19 case growth and morbidity and mortality rates [74]. Other scholars have studied the relationship between emotions and reposting behavior in the context of COVID-19, and Sun et al. found that posts with negative emotions related to the COVID-19 vaccine were more likely to be reposted on social media, and special attention should be paid to such posts to mitigate the negative effects of the information epidemic [75].

Sentiment analysis enables real-time monitoring of public attitudes and behaviors toward the COVID-19 vaccine, which is important for implementing timely interventions and policies. Thus, this paper conducts sentiment analysis of COVID-19-vaccine-hesitation posts on social-media platforms to uncover the sentiment differences among various types of hesitation reason posts.

## 3. Methods

### 3.1. Data Collection

Sina Microblog, a large social-networking platform in China, was chosen as the data source for this study. In China, Sina Microblog is the most popular platform for information acquisition, sharing and dissemination [76], and COVID-19 has generated an explosion of attention and discussion on Sina Microblog. This platform is open, with user-generated content on the platform available for public access and broadcast [77,78], and users can retrieve textual content by searching for specified keywords within a defined date range [38]. The data on the Sina Microblog platform is fully public and freely downloadable. None of the data obtained from the Sina Microblog platform in this paper involves sensitive information and personal privacy, and the data are used for scientific research only, with no commercial use involved.

We searched the Sina-Microblog platform for posts on COVID-19-vaccine hesitancy, which were posted by people who were hesitant about the COVID-19 vaccine. We set two search strategies, as shown in Appendix A. One was using twenty-three related keywords for the search in the Sina-Microblog search engine, such as “COVID-19 Vaccine Allergy” and “Vaccine Compulsory”. The other was to collect posts directly under nine related topics, such as the topic name is “Common reasons for hesitation in COVID-19 vaccination”, “COVID-19 vaccine will not cause leukemia and diabetes”, etc. We accessed the platform via the official website and collected data using the pre-defined search strategy. The data was collected from posts made between 1 October 2021 and 1 October 2022, as well as various information (e.g., gender and date of birth, etc.) from the posters’ personal homepages.

Official accounts on the Sina-Weibo platform do not express vaccine hesitation, but rather encourage the public to get vaccinated as soon as possible, so only data from individual users were collected in this study. Due to possible overlap between posts collected by the two search strategies, the data were de-duplicated (based on the unique id of the Sina-Weibo user), and irrelevant text (including advertisements, support for COVID-19 vaccination, or hesitation about other vaccines) was removed. A total of 28,031 relevant posts were collected, and the total number of valid posts after data cleaning was 12,703; 15,328 invalid posts were deleted.

**Figure 1:**
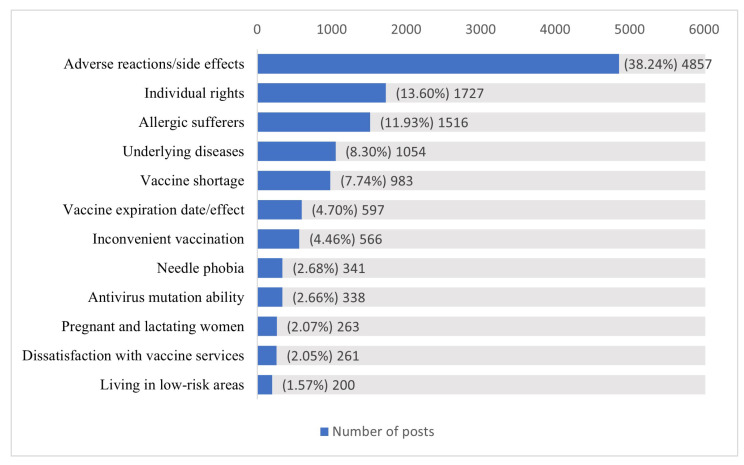
Distribution of the number of valid posts for 12 types of COVID-19-vaccine-hesitancy reasons (1 October 2021–1 October 2022).

Based on the automatic-identification model of reasons for COVID-19 vaccine hesitation and the twelve types of COVID-19 vaccine hesitation reasons model developed in previous studies, we classified the valid posts [79], in which 12,703 posts were, respectively, identified as one of the twelve types of vaccine-hesitation reasons. After classification, the distribution of the number of valid posts among the twelve types of COVID-19 vaccine hesitation reason is shown in Figure 1. The statistical results show that the number of posts in each type is very uneven. “Adverse reactions/side effects” are the dominant factors for COVID-19 vaccine hesitation, accounting for 38.24%. In addition to “Adverse reactions/side effects”, only two types of posts, “Individual rights” and “Allergic sufferers”, accounted for more than 10%.

### 3.2. User-Groups Analysis

**Figure 2:**
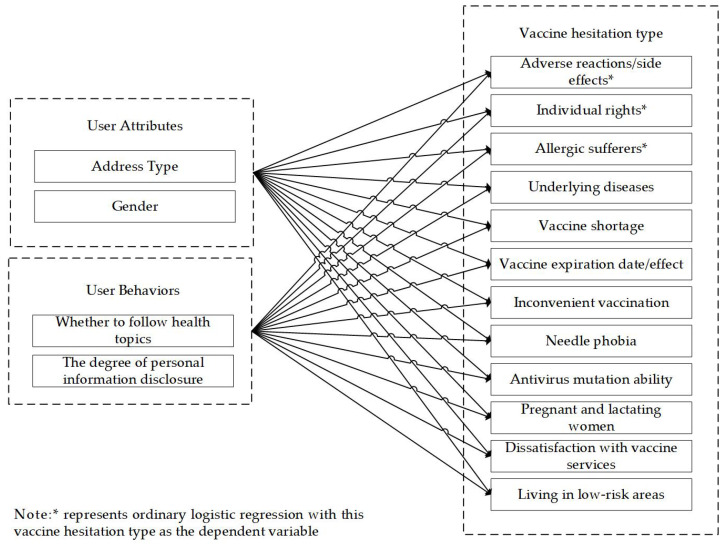
Research model for various types of vaccine-hesitator groups.

As shown in Figure 2, in this study, the independent variables include two parts: user attributes and user behaviors. Among them, user attributes include two variables: gender and address type; user behaviors include two variables: whether health topics are followed and the degree of personal-information disclosure. Among them, the degree of personal-information disclosure reflects the user’s willingness to disclose personal information on social media. Previous studies have shown that personal-information disclosure is an intentional behavior, reflecting the user’s internal psychological state and the willingness to establish external social relations with others, and is a manifestation of personal characteristics [80].

The research model includes twelve 0–1 dependent variables (dummy variables), namely, twelve different types of vaccine hesitation. The user-groups analysis used logistic regression to analyze the differences in user characteristics between the various types of posts. Logistic regression is a classical method commonly used to solve dichotomous classification problems and is widely used in the field of data mining. In addition, the data category-imbalance problem can make the model underestimate the incidence of fewer samples, which affects the effectiveness of the model. The rare-event logistic regression proposed by Gary King and Langche Zeng [81] can solve the category-imbalance problem, and the application scenario of this model is generally when the proportion of positive and negative samples is below 10% [82]. Therefore, three general logistic regression models were constructed with “Adverse reactions/side effects”, “Individual rights”, and “Allergic sufferers” (the top-three sample sizes with all positive samples being greater than 10%) as dependent variables, respectively, and the remaining types (all with positive sample proportions less than 10%) used rare-event logistic regression.

The types of independent variables and measurement criteria are shown in the Table 1. All four independent variables were obtained from the personal home pages of Sina-Microblog users. On the user’s information page, users are allowed to fill in the items of their choice from date of birth, horoscope, address, and profile (non-required items), so some users choose not to disclose a particular type of personal information. In this paper, the degree of personal-information disclosure is defined as a multicategorical variable. In addition to personal information, the homepage also shows various super-talkers (a circle formed by a collection of people with common interests) and topics that the user follows, so it is possible to identify whether the user follows health topics such as cervical spondylosis, migraine, hives, depression, health and wellness, etc.

All twelve dependent variables are defined as 0–1 dummy variables, with 0 indicating that the post does not belong to that category of vaccine-hesitation type and 1 indicating that the post belongs to that category of vaccine-hesitation type. For example, for the dependent variable vaccine shortage, this paper defines the posts marked as vaccine-shortage type as 1 and the rest of the posts as 0.

### 3.3. Text Sentiment Analysis

The sentiment-analysis tool in this study uses the Baidu Sentiment Tendency Analysis API, which is a sentiment-analysis tool with good results based on the Bi-LSTM model developed to fully understand the sentiment of posts by combining the semantics of text contexts. The platform also provides a customized version of the sentiment-analysis API to help users to upload their scenario-specific corpus to improve the accuracy of the model. Therefore, 700 texts with positive sentiment and 1000 texts with negative sentiment were added to the Baidu sentiment-analysis model for training and optimization (1700 texts were generated by random sampling, text data from previous studies [79], and all were manually labeled with sentiment polarity). Finally, the accuracy rate was improved to 91.76%. Subsequently, the trained model was called to perform sentiment analysis on 12,703 posts, and for each post, the model was able to identify the sentiment score and sentiment polarity of the post. The sentiment score takes the value of [0, 1], and the sentiment polarity includes three values: positive, neutral and negative. Specifically, the sentiment score and sentiment polarity correspond to each other. The negative sentiment of a post means that the score is between [0, 0.45), neutral is [0.45, 0.55), and positive is [0.55, 1], which is the classification threshold determined by the developers of Baidu Sentiment Tendency Analysis API.

## 4. Results

### 4.1. User-Groups Analysis Results

The logistic regression model has to consider the multicollinearity between independent variables. A commonly used measure to detect collinearity is the variance inflator factor (VIF), which is the ratio of the variance in the presence of multicollinearity between explanatory variables to the variance in the absence of multicollinearity. It is commonly defined as: VIF$\left(i\right)=1/\left(1-{R_{i}}^{2}\right)$, where ${R_{i}}^{2}$ is the coefficient of determination of the regression on the other independent variables with $x_{i}$ as the explanatory variable. Usually, when 0 < VIF < 10, there is no multicollinearity [83,84].

**Figure 3:**
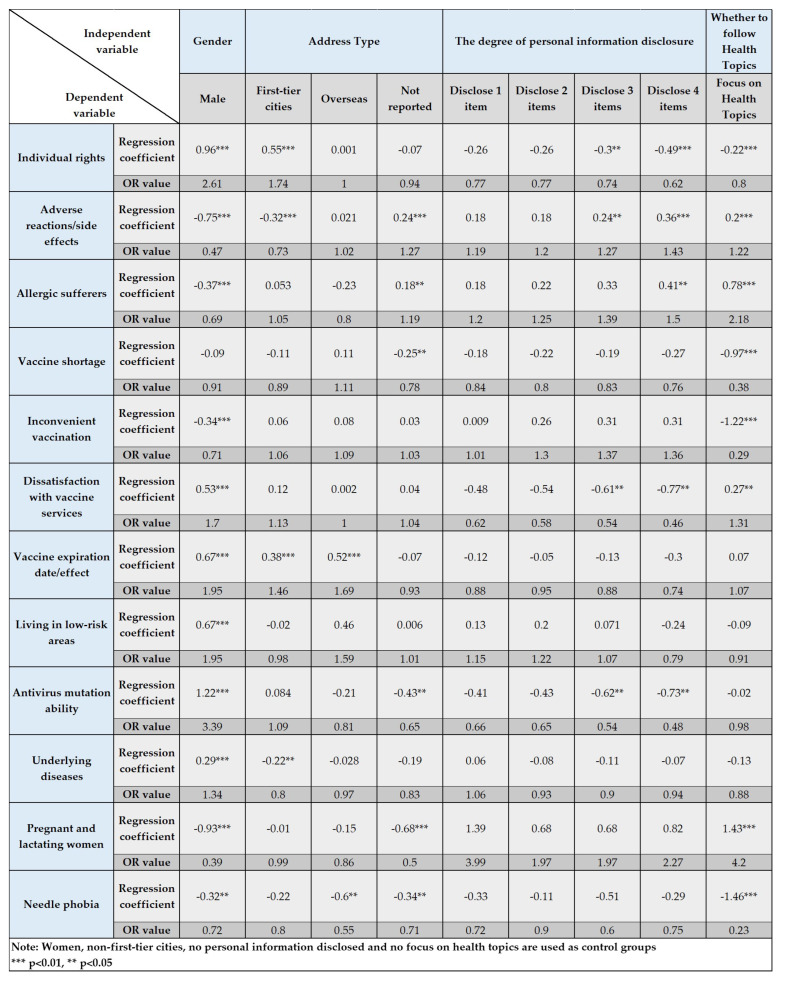
Regression model results.

The VIF values were calculated to determine whether there is a multicollinearity problem among the variables, and the test results are shown in Table 2. The VIF values of all variables are less than 1.5, so there is no multicollinearity. The results of the regression model are shown in Figure 3. The regression coefficients, p-values and OR values are reported in the table. The OR value (Odds ratio), also known as the dominance ratio, indicates the ratio between the probability of an event in the experimental group and the probability of an event in the control group. In logistic regression analysis, OR values are more explanatory than regression coefficients. Female, non-first-tier cities, no personal information disclosed, and no focus on health topics were used as controls, *** *p* < 0.01, ** *p* < 0.05.

#### 4.1.1. Differential Analysis of User Gender

The user gender sample is 73% female (9200) and 37% male (3502). Among the types of “Individual rights”, “Dissatisfaction with vaccine services”, “Vaccine expiration date/effect”, “Living in low-risk areas”, “Antivirus mutation ability”, and “Underlying diseases”, male users are significantly more likely to post these types of posts than female users. Males are 2.61 times more likely than females to publish vaccine-hesitation posts about “Individual rights” (*p* < 0.01); males are 1.7 times more likely than females to publish vaccine-hesitation posts about “Dissatisfaction with vaccine services” (*p* < 0.01); males are 1.95 times more likely than females to publish vaccine-hesitation posts about “Vaccine expiration date/effect” and “Living in low-risk areas” (*p* < 0.01); males are 3.39 times more likely than females to publish vaccine-hesitation posts about “Antivirus mutation ability” (*p* < 0.01); and males are 1.34 times more likely than females to publish vaccine hesitation posts about “Underlying diseases”(*p* < 0.01).

Concerning the five types of “Adverse reactions/side effects”, “Allergic sufferers”, “Inconvenient vaccination”, “Pregnant and lactating women” and “Needle phobia”, female users are significantly more likely to post than male users. Among them, the probability of males publishing vaccine-hesitation posts about “Adverse reactions/side effects” is 47% of females who post such positions (v0.01); the probability of males publishing vaccine-hesitation posts about “Allergic sufferers” is 69% of females (*p* < 0.01); the probability of males publishing vaccine-hesitation posts about “Inconvenient vaccination” is 71% of females (*p* < 0.01); the probability of males publishing vaccine-hesitation posts about “Pregnant and lactating women” is 39% of females (*p* < 0.01); and the probability of males publishing vaccine-hesitation posts about “Needle phobia” is 72% of females (*p* < 0.05).

#### 4.1.2. Differential Analysis of User Address Types

In the analysis of differences in user address types, we only focus on the first-tier cities and non-first-tier cities. The sample size of first-tier cities is 2667, and the sample size of non-first-tier cities is 6028. Users in first-tier cities are significantly more than users in non-first-tier cities for the two vaccine-hesitation types of “Individual rights” and “Vaccine expiration date/effect”. First-tier city users are 1.74 times more likely to post vaccine-hesitation posts about “Individual rights” than non-first-tier city users (*p* < 0.01), and first-tier city users are 1.46 times more likely to post vaccine-hesitation posts about “Vaccine expiration date/effect” than non-first-tier city users (*p* < 0.01).

In the two vaccine-hesitation types of “Adverse reactions/side effects” and “Underlying diseases”, users in non-first-tier cities are significantly more than users in first-tier cities. The probability of users in first-tier cities posting “Adverse reactions/side effects” vaccine-hesitation posts is 73% of those who posted such posts in non-first-tier cities (*p* < 0.01), and the probability of users in first-tier cities posting “Underlying diseases” vaccine-hesitation posts is 80% of those who posted such posts in non-first-tier cities (*p* < 0.01).

#### 4.1.3. Differential Analysis of the Degree of Personal-Information Disclosure

The sample size of those who do not disclose any personal information is 516 (4%), the sample size of those who disclose 1 piece of personal information is 1457 (11%), the sample size of those who disclose 2 pieces of personal information is 2407 (19%), the sample size of those who disclose 3 pieces of personal information is 4014 (32%), and the sample size of those who disclose 4 pieces of personal information is 4309 (34%). Users with high degrees of personal-information disclosure are more prone to post vaccine-hesitation posts about “Adverse reactions/side effects” and “Allergic sufferers” than users with low degrees of personal-information disclosure. In the type of “Adverse reactions/side effects”, users who disclosed 3 items of personal information are 1.27 times more likely than those who do not disclose any personal information (*p* < 0.05), and users who disclosed 4 items of personal information are 1.43 times more likely than those who do not disclose any personal information (*p* < 0.01). In the type of “Allergic sufferers”, users who disclosed 4 items of personal information are 1.5 times more likely than those who do not disclose any personal information (*p* < 0.05).

Users with low degrees of personal-information disclosure are more likely to post posts related to the types of “Individual rights”, “Dissatisfaction with vaccine services” and “Antivirus mutation ability” than users with high degrees of personal-information disclosure. In the type of “Individual rights”, the probability of users who disclosed 3 items of personal information is 74% of users who do not disclose any personal information (*p* < 0.05); the probability of users who disclosed 4 items of personal information is 62% of users who do not disclose any personal information (*p* < 0.01). In the type of “Dissatisfaction with vaccine services”, the probability of users who disclosed 3 items of personal information is 54% of users who do not disclose any personal information (*p* < 0.05); the probability of users who disclosed 4 items of personal information is 46% of users who do not disclose any personal information (*p* < 0.05). In the type of “Antivirus mutation ability”, the probability of users who disclosed 3 items of personal information is 54% of users who do not disclose any personal information (*p* < 0.05); the probability of users who disclosed 4 items of personal information is 48% of users who do not disclose any personal information (*p* < 0.05).

#### 4.1.4. Differential Analysis of Whether to Follow Health Topics

Among the sample of following health topics, 63% (7956) of users do not follow health topics and 37% (4747) of users follow health topics. Among the “Adverse reactions/side effects”, “Allergic sufferers”, “Dissatisfaction with vaccine services” and “Pregnant and lactating women”, users who followed health topics are significantly more than those who do not. In vaccine-hesitation posts about “Adverse reactions/side effects”, users who followed health topics are 1.22 times more than those who do not follow health topics (*p* < 0.01). In vaccine-hesitation posts about “Allergic sufferers”, users who followed health topics are 2.18 times more than those who do not follow health topics (*p* < 0.01). In vaccine-hesitation posts about “Dissatisfaction with vaccine services”, users who followed health topics are 1.31 times more than those who do not follow health topics (*p* < 0.05). In vaccine-hesitation posts about “Pregnant and lactating women”, users who followed health topics are 4.20 times more than those who do not follow health topics (*p* < 0.01).

Among “Individual rights”, “Vaccine shortage”, “Inconvenient vaccination” and “Needle phobia”, users who do not follow health topics are significantly more than those who do. In vaccine-hesitation posts about “Individual rights”, the probability of users who followed health topics is 80% of those who do not follow health topics (*p* < 0.05). In vaccine-hesitation posts about “Vaccine shortage”, the probability of users who followed health topics is 38% of those who do not follow health topics (*p* < 0.05). In vaccine-hesitation posts about “Inconvenient vaccination”, the probability of users who followed health topics is 29% of those who do not follow health topics (*p* < 0.05). In vaccine-hesitation posts about “Needle phobia”, the probability of users who followed health topics is 23% of those who do not follow health topics (*p* < 0.05).

Combining the differences in user gender, user address, user’s degree of personal-information disclosure, and whether users follow health topics, we found that there are differences in the types of COVID-19-vaccine-hesitation posts posted by users with different characteristics, and the relevance of the type of vaccine hesitation to the body becomes a delineating factor. According to common sense, we can consider “Underlying diseases”, “Needle phobia”, “Adverse reactions/side effects”, “Allergic sufferers”, “Pregnant and lactating women” as body-related types of vaccine hesitations, and “Individual rights”, “Inconvenient vaccination”, “Vaccine shortage”, “Dissatisfaction with vaccine services”, “Living in low-risk areas”, “Vaccine expiration date/effect”, “Antivirus mutation ability”, as non-body-related types of vaccine hesitations.

Based on the results, it can be briefly concluded that female users are more likely to post body-related vaccine-hesitation-type posts than male users; users in non-first-tier cities are more likely to post body-related vaccine-hesitation-type posts than users in first-tier cities; users with a high a degree of personal-information disclosure are more likely to post body-related vaccine-hesitation-type posts than users with a low degree of personal-information disclosure; and users who follow health topics are more likely to post body-related vaccine-hesitation-type posts than users who do not follow health topics. A more specific analysis follows in the discussion section.

### 4.2. Text Sentiment-Analysis Results

**Figure 4:**
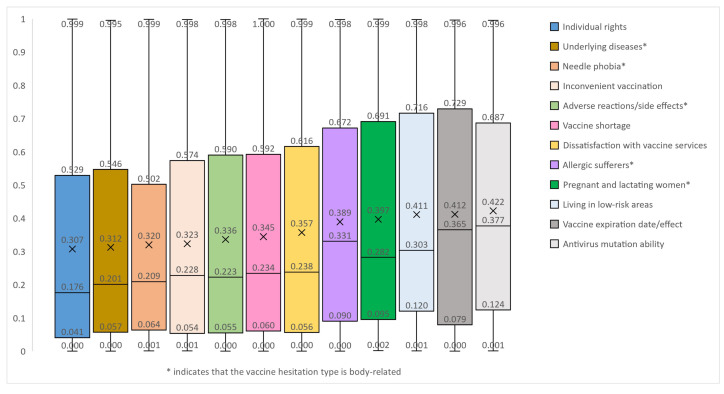
The average sentiment scores of posts with different vaccine hesitation types.

The average sentiment scores of vaccine-hesitation posts in twelve types are shown in Figure 4. As seen in the figure, the sentiment score for each post in each type of vaccine hesitation takes a value between [0, 1], and the average sentiment scores of posts in all twelve types are below 0.45. Meanwhile, the average affective score for the “Individual rights” type is the lowest among the twelve types with only 0.307. Among the types related to the body, the average score of posts of the “Underlying disease” type is the lowest, at only 0.312. In contrast, posts in the categories of “Living in low-risk areas”, “Vaccine expiration date/effect”, and “Antivirus mutation ability” have higher average sentiment scores of more than 0.4, all three types of vaccine hesitation are not relevant to the body.

**Figure 5:**
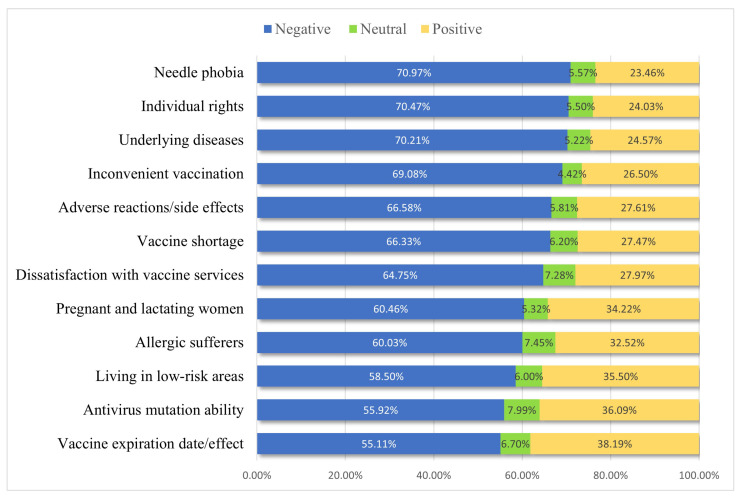
The distribution of sentiment polarity of posts with different vaccine-hesitation types.

The distribution of sentiment polarity of vaccine-hesitation posts in the twelve types is shown in Figure 5. Sentiment polarity is determined based on the sentiment score, which consists of three values: positive, neutral and negative. The post score between [0, 0.45) indicates the sentiment polarity of this post is negative, the score between [0.45, 0.55) indicates neutral sentiment polarity, and the score between [0.55, 1] indicates positive sentiment polarity. For example, in the “Needle phobia” type, nearly 71% of posts scored between [0, 0.45) and are judged to be negative affective posts, 5.6% of posts scored between [0.45, 0.55) and are judged to be neutral affective posts, and 23.4% of posts scored between [0.55, 1] and are judged to be positive affective posts.

As a whole, the percentage of negative posts of all vaccine-hesitation types is distributed around 55% to 70%. The proportion of negative posts is distributed between about 55% and 70%. The three categories that accounted for more than 70% of the posts are “Needle phobia”, “Individual rights” and “Underlying disease”, with the highest proportion being “Needle phobia”. Neutral posts accounted for a small percentage in all categories, all less than 8%. Positive posts accounted for about 23% to about 38%, with the highest proportion of posts in the three categories of “Vaccine expiration date/effect”, “Antivirus mutation ability”, and “Living in low-risk areas”, consistent with the results of the average sentiment score. The proportion of negative sentiment is the largest in each type, indicating that each type of vaccine-hesitation type post is rather negative.

To further test whether the sentiment scores of the twelve vaccine-hesitation-type posts were statistically significantly different, we first performed a Kolmogorov–Sminov normality test on the twelve vaccine-hesitation-type posts. The Kolmogorov–Smirnov test is a nonparametric test that is generally applied to large samples, and the sample size of the vaccine-hesitation post in this paper is 28,031, so it is applicable. The original hypothesis is that the sample obeys a normal distribution. If the significance is less than 0.05, the original hypothesis is rejected, that is, the sample does not obey the normal distribution; if the significance is greater than 0.05, the original hypothesis is accepted, that is, it obeys the normal distribution. The test results are shown in Table 3, the significance of each group of samples is less than 0.05, so they do not obey the normal distribution.

Analysis of variance (ANOVA) is a commonly used parametric method for testing differences in means between more than two groups, but its limitation is the assumption of normality, which makes ANOVA very inefficient under the influence of non-normal data distribution [85]. The Kruskal–Wallis (K-W) test can be used when the sample data does not conform to a normal distribution and the data is continuous and independent [86]. Since the samples in this study are non-normally distributed and the dependent variable is continuous, the (Kruskal–Wallis test is) used to test whether multiple independent samples came from the same probability distribution. The original hypothesis is that the distribution of sentiment scores of posts is the same in each type of vaccine hesitation. If the significance is less than 0.05, it indicates that there is a significant difference between multiple samples. The significance of the K–W test in this study is less than 0.05. Therefore, the original hypothesis is rejected, which means the sentiment scores of the twelve types of vaccine-hesitation posts are significantly different. Further, a two-by-two comparison between each sample allows for the observation of whether there is a significant difference between every two samples. Table 4 shows the results of the two-by-two comparisons and, due to the large number of types, only the sample pairs with significant differences are reported in the table (*p*-value < 0.05).

The results showed that the sentiment score of posts in the “Individual rights” type is significantly lower than those in the other seven types. The sentiment score of posts in the “Inconvenient vaccination” type is significantly lower than those in the four types. The sentiment score of posts in the “Underlying diseases” type is lower than those in the five types. The sentiment scores of posts in the “Adverse reactions/side effects” and “Needle phobia” types are lower than those in the four and three types, respectively. By logical deduction, it can be concluded that the lower sentiment score group includes five types: “Individual rights”, “Inconvenient vaccination”, “Underlying diseases”, “Adverse reactions/side effects” and “Needle phobia”, among which the most negative sentiment is the “Individual rights”. The higher sentiment score group is “Vaccine expiration date/effect”, “Living in low-risk areas”, and “Antivirus mutation ability”.

**Figure 6:**
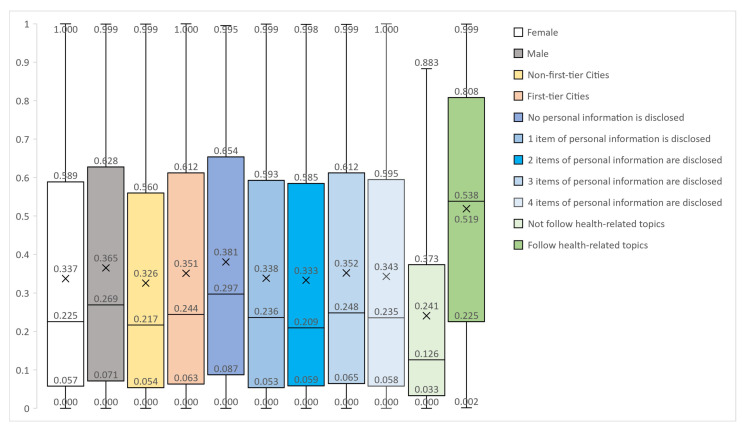
The average sentiment scores with different user characteristics.

**Figure 7:**
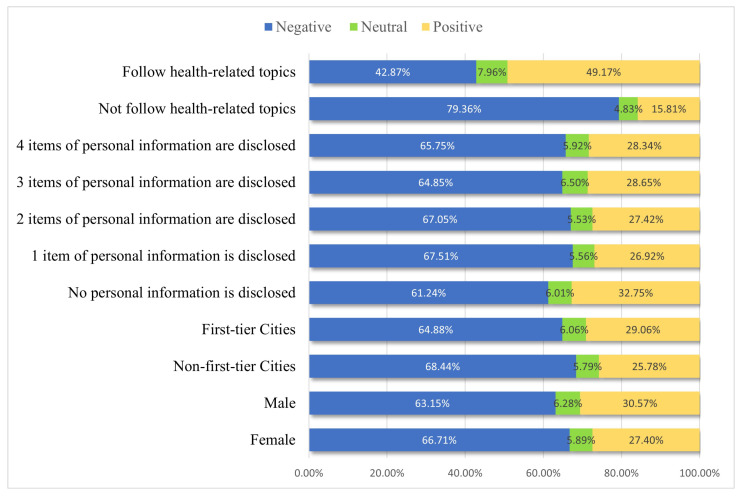
The distribution of sentiment polarity of posts with different user characteristics.

Based on the sentiment scores of all posts, sentiment differences can also be analyzed from four perspectives: gender, type of address, whether to follow health topics, and degree of personal-information disclosure. The following conclusions can be drawn by combining Figure 6 and Figure 7.

In terms of gender, both male and female users have an average sentiment score of less than 0.4. Both the proportions of negative posts is the highest, about 63% to 67%. Neutral posts account for the least amount of posts, both less than 6.5%. The proportion of positive posts is around 27% to 31%.

The average sentiment score for users at different address types and with different degrees of personal-information disclosure is also below 0.4. The proportion of negative posts is the highest, about 61% to 67%. Neutral posts account for the fewest posts, all less than 6.5%. The proportion of positive posts ranges from 25% to 29%. Gender, address type, and degree of personal-information disclosure are similar in the distribution of sentiment scores and sentiment polarity, with users of different user characteristics having relatively negative sentiments.

There is a large difference in sentiment in terms of whether or not usesr follow health topics. Users who followed health topics have an average sentiment score above 0.5, and the proportion of positive posts is the highest (about 49%), while users who do not follow health topics have an average sentiment below 0.25, and the proportion of negative posts is the highest (79.4%). This indicates that users who have followed health topics are likely to be more positive and those who have not followed health topics are likely to be more negative.

## 5. Disscusson

This paper constructs user personas of 12,703 COVID-19 vaccine hesitators on social media in terms of gender, address type, whether they follow health topics, and degree of personal-information disclosure, and the results show that there are differences in user attributes and user behaviors among different COVID-19 vaccine-hesitation types. Users with different characteristics have different reasons for hesitating about the COVID-19 vaccine, indicating that users with different characteristics have different concerns about the COVID-19 vaccine. The results can, therefore, be used to reduce the public’s vaccine hesitation by taking advantage of the big-data precision push of social-media platforms to push targeted and relevant information.

In terms of user gender, of the types of “Individual rights”, “Dissatisfaction with vaccine services”, “Vaccine expiration date/effect”, “Living in low-risk areas”, “Antivirus mutation ability”, and “Underlying diseases”, male users are significantly more likely to post these types of posts than female users. It can be found that except for the “Underlying diseases”, the reasons for vaccine hesitation among male users are mainly non-physical-related factors, more often considering whether vaccination against COVID-19 is necessary. This gender difference is particularly prominent in the “Individual rights” due to the predominantly self-oriented nature of men [87]. Posts of the “Individual rights” type usually express the user’s resentment and resistance to vaccination due to certain forced vaccination practices or requirements. Psychological studies have shown that women have better self-control in the face of disliked or repulsive behaviors. In contrast, men are more impulsive and adventurous [88] and more likely to develop strong resistance when encountering coercive and compulsive situations.

In the five types of “Adverse reactions/side effects”, “Allergic sufferers”, “Inconvenient vaccination”, “Pregnant and lactating women” and “Needle phobia”, female users are significantly more likely to post these types of posts than male users. It can be found that except for the type of “Inconvenient vaccination”, the reasons for vaccine hesitation among female users are all factors closely related to the body. Previous studies have shown that females experience more adverse reactions after vaccination compared to males [89], and gender differences in vaccine side effects have been observed not only in COVID-19 vaccination [90] but also widely in BCG, measles, the yellow fever virus vaccine and the influenza vaccine. The prevalence of needle phobia is higher in females, commonly occurs in the pediatric population, and decreases with age [91]. In addition, women, who are generally the more responsible party in the family, are often the key decision-makers on whether to vaccinate the children and elderly in the family [92] and need to coordinate the planning of their own and their family’s vaccination schedule and location, “Inconvenient vaccination” is more likely to be a deterrent to vaccination in women. There is no significant difference by gender in “Vaccine shortage”. This is because vaccine shortage is an objective situation that puts vaccinators in a completely passive position due to lack of capacity or epidemic closure and does not change with the subjective thoughts of people, and, therefore, is not affected by gender.

In terms of user address types, we focus only on the differences between first-tier and non-first-tier cities. Users in first-tier cities are significantly more than users in non-first-tier cities for the two vaccine-hesitation types of “Individual rights” and “Vaccine expiration date/effect”.The proportion of higher education groups in first-tier cities is high. On the one hand, such groups are better at self-expression, are more aware of and defend their rights, and are more willing to speak out on public issues; on the other hand, the more educated groups are also more inclined to think rationally and are more likely to understand and consider the effectiveness and effective duration of vaccines; this is also verified in a previous study [18].

In the two vaccine-hesitation types of “Adverse reactions/side effects” and “Underlying diseases”, users in non-first-tier cities are significantly more than users in first-tier cities. The occurrence of adverse reactions and the underlying disease of the vaccine recipient themself is very common in all regions, and the population size in non-first-tier cities is much larger than in first-tier cities; thus, the difference in the type of address between these two types on social platforms is reasonable.

In terms of the degree of disclosure of personal information, users with high degrees of personal-information disclosure are more prone to post vaccine-hesitation posts about “Adverse reactions/side effects” and “Allergic sufferers” than users with low degrees of personal-information disclosure. The degree of personal-information disclosure reflects the willingness of that user to disclose personal information. In the type of “Adverse reactions/side effects” and “Allergic sufferers”, it is necessary to describe the user’s symptoms and physical condition, which is also more private personal information. Hence, users who are willing to post these two types of posts are more likely to have a higher willingness to disclose personal information.

Users with low degrees of personal-information disclosure are more likely to post posts related to “Individual rights”, “Dissatisfaction with vaccine services” and “Antivirus mutation ability” than users with high degrees of personal information disclosure. These three vaccine-hesitation types have nothing to do with the user’s body, and the posts do not involve the disclosure of the personal privacy of the user.

In terms of following health topics, among the “Adverse reactions/side effects”, “Allergic sufferers”, “Dissatisfaction with vaccine services” and “Pregnant and lactating women”, users who followed health topics are significantly more than those who do not. Except for “Dissatisfaction with vaccine services”, the other three types of vaccine hesitancy are all factors closely related to the body. The behavior of following health topics on social-media platforms reflects the importance users place on their health. When health-conscious users are faced with the decision of whether to receive the COVID-19 vaccine, they carefully consider whether their health condition is suitable for the vaccine and the potential adverse reactions from the vaccine. Previous studies have also shown that people with pre-existing conditions and those who are concerned about their health are more hesitant about vaccines [93]. In addition to this, health-conscious users are likely to have higher expectations of healthcare services, and, therefore, significant differences emerge in the “Dissatisfaction with vaccine services”.

Among “Individual rights”, “Vaccine shortage”, “Inconvenient vaccination” and “Needle phobia”, users who do not follow health topics are significantly more than those who do. Except for “Needle phobia”, the other three types of vaccine hesitancy are not related to the body. In contrast, in some studies, people with needle phobia had lower adherence to medical treatment, and not following health topics on social platforms could also be seen as a form of medical avoidance for people with needle phobia [94].

The relevance of the reason for vaccine hesitancy to the body became a delineating factor when mining the differences in user characteristics. Therefore, based on the differences in user characteristics in the four dimensions of gender, address type, degree of personal disclosure, and whether users followed health topics, we can present two COVID-19 vaccine-hesitant user groups, Body-related and Non-bodily-related. The body-related user group tends to be female, has a higher disclosure of personal information, and follows health topics on social-media platforms. The non-bodily-related user group tends to be male, has a low disclosure of personal information, and does not follow health topics on social-media platforms.

Body-related users post more about adverse reactions, allergic reactions and pregnancy reactions. They are more health-conscious and have more concerns and worries that vaccination may induce diseases that can cause irreversible damage to their bodies. Therefore, one of the strategies to alleviate the vaccine hesitation of this group of users is to use social-media platforms to push relevant scientific awareness content to highlight the safety of the COVID-19 vaccination to them and alleviate their excessive concerns about their health conditions. For example, COVID-19 vaccination is safe and effective in pregnant and lactating people, and antibodies appear in breast milk to protect infants from COVID-19 after the vaccination of lactating mothers [95,96]. Individuals with a history of specific allergies or severe reactions to the vaccine may be tested dermally and in vitro using the vaccine or other components [97]. On the other hand, the level of vaccine science articles on the internet varies and their scientific views are inconsistent; for example, some articles suggest that pregnancy is not a contraindication to COVID-19 vaccination, while others suggest that pregnant people should not be vaccinated, which may make body-related users more hesitant and anxious. There are also instances of different policy implementations online and offline. In the vaccination guidelines published by leading experts, most underlying conditions that are not in acute exacerbation are not contraindications to COVID-19 vaccination [98,99]. However, some healthcare professionals at offline vaccination sites have indicated that a certain condition makes an individual not suitable for vaccination. Therefore, strategy two is to have experts from authoritative public-health institutions work together to determine whether the COVID-19 vaccine can be administered to specific populations in specific situations and to remove unreliable scientific articles from the Internet.

Non-bodily-related users post more about individual rights, vaccine services or propaganda, and the quality and safety of vaccines. social-media platforms can, therefore, prioritize the promotion of the principle of voluntary vaccination, positive publicity about vaccine services, the expiry date of vaccines, antiviral mutations and other relevant research reports to these users’ pages. Concerning the service and promotion of vaccination, it is important to optimize the vaccine supply system and improve the level of vaccination services, for example, by focusing on the training of their health-care professionals and improving their professionalism and attitude to service. People seem to have different preferences regarding the promotion of vaccination, with some people promoting their vaccination behavior through the use of humorous slogans, while others do the opposite [100]. The way in which vaccination is promoted should, therefore, be tailored to the specific situation. Ensuring the quality and safety of the COVID-19 vaccine itself is of paramount importance to increasing the public’s willingness to be vaccinated. Due to viral variation, immunologists believe that the resistance to viral variation and the duration of effectiveness of the neostriatal vaccine are inconclusive [101] and need to be evaluated in the context of future long-term clinical data. As the COVID-19 pandemic continues, public-health agencies should continue to track the overall incidence of adverse reactions and corresponding symptoms and collaborate with social media and social platforms to release timely and long-term reports of adverse reactions to the COVID-19 vaccine and data on vaccine efficacy and safety to the public.

In addition, this paper used Baidu Sentiment Tendency Analysis API to perform sentiment analysis on all posts. The average sentiment scores and the distribution of sentiment polarity of twelve types of COVID-19-vaccine-hesitation posts were counted. The sentiment scores of the twelve types of vaccine-hesitation-type posts were verified to be significantly different by the K–W non-parametric test. The average sentiment scores of all types of COVID-19-vaccine-hesitation posts were less than 0.45, and the proportion of negative sentiment was the largest for all types, with the proportion of negative posts distributed around 55% to 70%. The most negative sentiment is the “Individual rights” type. The higher sentiment scores are “Vaccine expiration date/effect”, “Living in low-risk areas” and “Antivirus mutation ability”. Additional sentiment analysis was conducted from four perspectives: gender, type of address, whether users follow health topics and degree of personal-information disclosure. It is found that users who follow health topics are likely to be more positive, and those who do not follow health topics are likely to be more negative. However, there is no significant difference in sentiment on the other three user characteristics, which are all more negative.

It can be seen that the emotions in each type of COVID-19 vaccine hesitant posts are more negative. Vaccine hesitancy is a psychological state of resistance or hesitation to vaccination, and vaccine hesitation individuals have some degree of negative emotions such as worry, fear, and resistance to vaccination or the vaccine itself. Previous studies have also found that social media texts about the COVID-19 vaccine are mostly dominated by negative emotions [70,73,74]. The “Individual rights” is the most negative of all types, as posts in this type are usually expressed as the act or requirement of being forced to receive the vaccine, which can cause users to feel antipathy and resistance to vaccination. Therefore, for this group, the principle of voluntary vaccination should be adhered to in order to avoid the formation of anti-vaccine groups that pose a greater public-health risk as a result of compulsory vaccination.

## 6. Conclusions

This paper used text-mining techniques to obtain COVID-19-vaccine-hesitant posts and user characteristics of these posters from the Sina-Microblog platform. A pre-developed classification model was then used to identify all posts for vaccine-hesitation types. Conducting user-groups analysis on COVID-19 vaccine hesitators identified the differences in user characteristics behind different hesitation-reason texts. Sentiment analysis was used on COVID-19-vaccine-hesitation posts to find out the sentiment differences of various types of hesitation reason posts. The user groups results show differences in both user attributes and user behaviors across COVID-19 vaccine hesitation types and correlation with the body, Ultimately two COVID-19 vaccine-hesitant user groups were presented: body-related and non-bodily-related. The results of the sentiment analysis show that the average sentiment score for all COVID-19 vaccine-hesitant-type posts is less than 0.45, and the sentiment of posts in each type is relatively negative. The “Individual rights” type is the most negative.

### 6.1. Academic Contribution

In this study, data were obtained from large social-networking platforms and a research model of the COVID-19-vaccine-hesitant cohort was constructed using a mixture of ordinary logistic regression and rare logistic regression. The population characteristics of vaccine hesitators were explored and, finally, divided into two major groups, which provide relevant theories and research methods for subsequent studies on COVID-19 vaccine hesitancy. This research framework is applicable to the analysis of vaccine-hesitant user groups on social platforms extended to different countries. Currently, there are relatively few studies that use social platforms to study the attribute characteristics and behavioral features of COVID-19 vaccine-hesitant groups. In addition, our study complements the application of user groups in the field of vaccine hesitancy, where user groupings are used as part of developing personas, and the results of the classification of user groups in this paper can provide relevant references for developing user personas for vaccine hesitancy.

### 6.2. Practical Significance

The results of this study on group characteristics and affective differences can contribute to an in-depth and comprehensive analysis of the needs of COVID-19 vaccine hesitators and help public-health agencies develop more targeted strategies to eliminate vaccine hesitancy, which has far-reaching implications for COVID-19 vaccine promotion and vaccination.

Based on the results of user-group analysis, the advantages of big-data-verified pushing of social platforms are used to push targeted and relevant information, to reduce the public’s hesitation about vaccines. For example, based on the gender differences found, relevant scientific information can be pushed for women whose reasons for hesitation are more physically related and for men who are more concerned about individual rights, services or publicity, vaccine effect or validity period, priority can be given to content such as research reports related to vaccine effectiveness and safety, posts that positively promote vaccine services, and posts that promote the principle of voluntary vaccination.

The results of the sentiment analysis showed that the sentiment of each category of COVID-19-vaccine-hesitation posts was more negative, indicating that the work related to COVID-19 vaccination needs to be further optimized and improved. The results of sentiment differences can provide directional references and suggestions for public-health agencies, such as the development of vaccine policies and immediate optimization of vaccine supply systems and vaccination services. For the “Individual rights” category, which has the most negative sentiment score, the voluntary principle of vaccination should be upheld to avoid the formation of anti-vaccine groups that are more harmful to public health due to mandatory vaccination.

### 6.3. Limitation and Future Work

Data for this study were obtained from the Chinese social-media platform Sina Microblog, but China has a relatively low ranking in media freedom (175/180) [102]; thus, there may be data bias in the data. The data collected is from only one platform and only spans one year, so the generalization ability of the model may be weak The classification model for vaccine-hesitant posts only used a pre-trained SVM classification model, and different classification models may cause differences in results. In future research, we can expand the period of data collection and use other vaccine-hesitation-type classification models to further verify the characteristics of COVID-19 vaccine hesitators and the emotional differences in various vaccine-hesitation posts. More user characteristics of COVID-19 vaccine hesitators can be collected to iterate user groups and abstract user personas from them. It is also possible to explore the sentiment differences between the user groups of COVID-19 vaccine hesitators and COVID-19-vaccine-hesitant posts from other social-media platforms for comparative analysis.

## Figures and Tables

**Table 1:** Types of independent variables and measurement criteria.

No.	Variable Name	Variable Type	Measurement Standard
1	Gender	0–1 variables	0 means female (9200); 1 means male (3503)
2	Address Type	Multicategorical variables	0 indicates non-first-tier cities (6028); 1 indicates first-tier cities (2667); 2 indicates overseas (881); 3 indicates not reported (3127)
3	The degree of personal information disclosure	Multi-categorical variables	0 means no personal information disclosed (516); 1 means 1 personal information is disclosed (1457); 2 means 2 items of personal information are disclosed (2407); 3 means that 3 items of personal information are disclosed (4014); 4 means that 4 items of personal information are disclosed (4309)
4	Whether to follow Health Topics	0–1 variables	0 means the user does not follow health-related topics (7956); 1 means that the user follows health-related topics (4747)

Note: The sample statistics of each variable are represented in parentheses.

**Table 2:** Results of multicollinearity test.

No.	Variable Name	VIF
1	Gender	1.01
2	Address type	1.43
3	The degree of personal-information disclosure	1.44
4	Whether to follow health topics	1.00
	Average VIF	1.22

**Table 3:** Results of Kolmogorov–Smirnov test.

Type	Statistics	Degrees of Freedom	Significance ^a^
Vaccine shortage	0.148	983	<0.001
Individual rights	0.167	1727	<0.001
Inconvenient vaccination	0.152	566	<0.001
Dissatisfaction with vaccine services	0.148	261	<0.001
Vaccine expiration date/effect	0.144	597	<0.001
Living in low-risk areas	0.137	200	<0.001
Antivirus mutation ability	0.107	338	<0.001
Underlying diseases	0.149	1054	<0.001
Adverse reactions/side effects	0.145	4857	<0.001
Pregnant and lactating women	0.147	263	<0.001
Needle phobia	0.153	341	<0.001
Allergic sufferers	0.112	1516	<0.001

Note: ^a^. Riley’s significance correction.

**Table 4:** Results of the Kruskal-Wallis test (two-by-two comparison).

Type	Comparison Type	Significance	Results of Two-Way Comparison (Emotional Score)
Individual rights	Vaccine shortage	0.013	Individual rights < Underlying diseases < Inconvenient vaccination, Adverse reactions/side effects < Needle phobia < Adverse reactions/side effects, Living in low-risk areas, Antivirus mutation ability
	Vaccine expiration date/effect	<0.001	
	Living in low-risk areas	<0.001	
	Antivirus mutation ability	<0.001	
	Adverse reactions/side effects	0.002	
	Living in low-risk areas	<0.001	
	Pregnant and lactating women	<0.001	
	Allergic sufferers	<0.001	
Inconvenient vaccination	Vaccine expiration date/effect	0.001	
	Living in low-risk areas	0.02	
	Antivirus mutation ability	<0.001	
	Allergic sufferers	<0.001	
Underlying diseases	Vaccine expiration date/effect	<0.001	
	Living in low-risk areas	0.003	
	Antivirus mutation ability	<0.001	
	Pregnant and lactating women	0.011	
	Allergic sufferers	<0.001	
Adverse reactions/side effects	Vaccine expiration date/effect	<0.001	
	Living in low-risk areas	0.027	
	Antivirus mutation ability	<0.001	
	Allergic sufferers	<0.001	
Needle phobia	Vaccine expiration date/effect	0.045	
	Antivirus mutation ability	0.003	
	Allergic sufferers	0.046	

**Figure A1:**
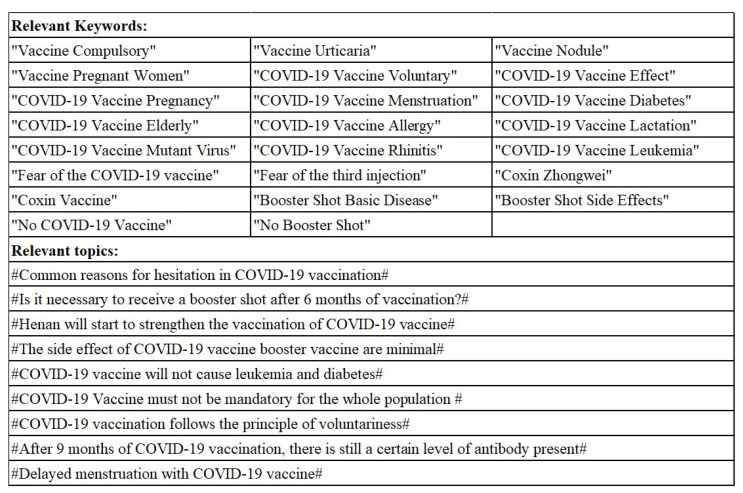
Search Strategies.

## Data Availability

Not applicable.

## References

[1] A Comprehensive Review of COVID-19 Characteristics|Biological Procedures Online|Full Text. https://biologicalproceduresonline.biomedcentral.com/articles/10.1186/s12575-020-00128-2.

[2] Coronavirus Disease (COVID-19). https://www.who.int/emergencies/diseases/novel-coronavirus-2019.

[3] Stasi C., Fallani S., Voller F., Silvestri C. (2020). Treatment for COVID-19: An Overview. Eur. J. Pharmacol..

[4] Ye X.T., Luo Y.L., Xia S.C., Sun Q.F., Ding J.G., Zhou Y., Chen W., Wang X.F., Zhang W.W., Du W.J. (2020). Clinical Efficacy of Lopinavir/Ritonavir in the Treatment of Coronavirus Disease 2019. Eur. Rev. Med. Pharmacol. Sci..

[5] Capalbo C., Aceti A., Simmaco M., Bonfini R., Rocco M., Ricci A., Napoli C., Rocco M., Alfonsi V., Teggi A. (2020). The Exponential Phase of the Covid-19 Pandemic in Central Italy: An Integrated Care Pathway. Int. J. Environ. Res. Public Health.

[6] Haji Abdolvahab M., Moradi-Kalbolandi S., Zarei M., Bose D., Majidzadeh-A K., Farahmand L. (2021). Potential Role of Interferons in Treating COVID-19 Patients. Int. Immunopharmacol..

[7] Yan Z., Yang M., Lai C.-L. (2021). Long COVID-19 Syndrome: A Comprehensive Review of Its Effect on Various Organ Systems and Recommendation on Rehabilitation Plans. Biomedicines.

[8] Ruggiero V., Aquino R.P., Del Gaudio P., Campiglia P., Russo P. (2022). Post-COVID Syndrome: The Research Progress in the Treatment of Pulmonary Sequelae after COVID-19 Infection. Pharmaceutics.

[9] (2021). Long COVID or Post COVID-19 Syndrome. Mult. Scler. Relat. Disord..

[10] Haeusermann T., Romero-Kornblum H., Dzeng E. (2022). Of Care, Cure and the in-between: COVID-19 Treatment in a New York City Intensive Care Unit. Int. J. Care Caring.

[11] Cazzola M., de Novellis V., Bianco A., Rogliani P., Matera M.G. (2021). Disputes over the Production and Dissemination of Misinformation in the Time of COVID-19. Respir. Med..

[12] Derosa G., Maffioli P., D’Angelo A., Di Pierro F. (2021). Nutraceutical Approach to Preventing Coronavirus Disease 2019 and Related Complications. Front. Immunol..

[13] Dubé E., Laberge C., Guay M., Bramadat P., Roy R., Bettinger J.A. (2013). Vaccine Hesitancy: An Overview. Hum. Vaccines Immunother..

[14] Wang B., Zhong X., Fu H., He M., Hu R. (2022). COVID-19 Vaccine Hesitancy and GAD: The Role of Risk Perception and Vaccination Status. Front. Public Health.

[15] Feikin D.R., Higdon M.M., Abu-Raddad L.J., Andrews N., Araos R., Goldberg Y., Groome M.J., Huppert A., O’Brien K.L., Smith P.G. (2022). Duration of Effectiveness of Vaccines against SARS-CoV-2 Infection and COVID-19 Disease: Results of a Systematic Review and Meta-Regression. Lancet.

[16] COVID-19 Vaccines Advice. https://www.who.int/emergencies/diseases/novel-coronavirus-2019/covid-19-vaccines/advice.

[17] Emary K.R.W., Golubchik T., Aley P.K., Ariani C.V., Angus B., Bibi S., Blane B., Bonsall D., Cicconi P., Charlton S. (2021). Efficacy of ChAdOx1 NCoV-19 (AZD1222) Vaccine against SARS-CoV-2 Variant of Concern 202012/01 (B.1.1.7): An Exploratory Analysis of a Randomised Controlled Trial. Lancet.

[18] Li Z., Ji Y., Sun X. (2022). The Impact of Vaccine Hesitation on the Intentions to Get COVID-19 Vaccines: The Use of the Health Belief Model and the Theory of Planned Behavior Model. Front. Public Health.

[19] Zeng B., Gao L., Zhou Q., Yu K., Sun F. (2022). Effectiveness of COVID-19 Vaccines against SARS-CoV-2 Variants of Concern: A Systematic Review and Meta-Analysis. BMC Med..

[20] Ma C., Sun W., Tang T., Jia M., Liu Y., Wan Y., Han J., Rodewald L., Li J., Song Y. (2022). Effectiveness of Adenovirus Type 5 Vectored and Inactivated COVID-19 Vaccines against Symptomatic COVID-19, COVID-19 Pneumonia, and Severe COVID-19 Caused by the B.1.617.2 (Delta) Variant: Evidence from an Outbreak in Yunnan, China, 2021. Vaccine.

[21] COVID-19 Vaccines. https://www.who.int/emergencies/diseases/novel-coronavirus-2019/covid-19-vaccines.

[22] Schaffer DeRoo S., Pudalov N.J., Fu L.Y. (2020). Planning for a COVID-19 Vaccination Program. JAMA.

[23] Chen Y.-T. (2021). The Effect of Vaccination Rates on the Infection of COVID-19 under the Vaccination Rate below the Herd Immunity Threshold. Int. J. Environ. Res. Public Health.

[24] Uzun O., Akpolat T., Varol A., Turan S., Bektas S.G., Cetinkaya P.D., Dursun M., Bakan N., Ketencioglu B.B., Bayrak M. (2022). COVID-19: Vaccination vs. Hospitalization. Infection.

[25] Lin X.-Q., Zhang M.-X., Chen Y., Xue J.-J., Chen H.-D., Tung T.-H., Zhu J.-S. (2022). Relationship between Knowledge, Attitudes, and Practices and COVID-19 Vaccine Hesitancy: A Cross-Sectional Study in Taizhou, China. Front. Med..

[26] Olagoke A.A., Olagoke O.O., Hughes A.M. (2021). Intention to Vaccinate Against the Novel 2019 Coronavirus Disease: The Role of Health Locus of Control and Religiosity. J. Relig. Health.

[27] Seddig D., Maskileyson D., Davidov E., Ajzen I., Schmidt P. (2022). Correlates of COVID-19 Vaccination Intentions: Attitudes, Institutional Trust, Fear, Conspiracy Beliefs, and Vaccine Skepticism. Soc. Sci. Med..

[28] Wang J., Ji Q., Dong S., Zhao S., Li X., Zhu Q., Long S., Zhang J., Jin H. (2021). Factors Influencing Vaccine Hesitancy in China: A Qualitative Study. Vaccines.

[29] Feng H., Zhu H., Zhang H., Cao L., Li L., Wang J., Huang Y., Lai X., Lyu Y., Jing R. (2021). Caregivers’ Intentions to COVID-19 Vaccination for Their Children in China: A Cross-Sectional Survey. Hum. Vaccine Immunother..

[30] Sun Y., Chen X., Cao M., Xiang T., Zhang J., Wang P., Dai H. (2021). Will Healthcare Workers Accept a COVID-19 Vaccine When It Becomes Available? A Cross-Sectional Study in China. Front. Public Health.

[31] Yang R., Penders B., Horstman K. (2020). Addressing Vaccine Hesitancy in China: A Scoping Review of Chinese Scholarship. Vaccines.

[32] Shen X., Dong H., Feng J., Jiang H., Dowling R., Lu Z., Lv C., Gan Y. (2021). Assessing the COVID-19 Vaccine Hesitancy in the Chinese Adults Using a Generalized Vaccine Hesitancy Survey Instrument. Hum. Vaccine Immunother..

[33] Qin C., Yan W., Tao L., Liu M., Liu J. (2022). The Association between Risk Perception and Hesitancy toward the Booster Dose of COVID-19 Vaccine among People Aged 60 Years and Older in China. Vaccines.

[34] Liu H., Zhou Z., Tao X., Huang L., Zhu E., Yu L., Zhang M. (2021). COVID-19 Vaccine Hesitancy among Chinese Residents under the Free Vaccination Policy. Rev. Assoc. Med. Bras..

[35] MacDonald N.E. (2015). Vaccine Hesitancy: Definition, Scope and Determinants. Vaccine.

[36] Reticencia a La Vacunación: Un Desafío Creciente Para Los Programas de Inmunización. https://www.who.int.

[37] Zhu J., Weng F., Zhuang M., Lu X., Tan X., Lin S., Zhang R. (2022). Revealing Public Opinion towards the COVID-19 Vaccine with Weibo Data in China: BertFDA-Based Model. Int. J. Environ. Res. Public Health.

[38] Wang J., Zhou Y., Zhang W., Evans R., Zhu C. (2020). Concerns Expressed by Chinese Social Media Users During the COVID-19 Pandemic: Content Analysis of Sina Weibo Microblogging Data. J. Med. Internet Res..

[39] Larson H.J., Gakidou E., Murray C.J.L. (2022). The Vaccine-Hesitant Moment. N. Engl. J. Med..

[40] Peretti-Watel P., Larson H.J., Ward J.K., Schulz W.S., Verger P. (2015). Vaccine Hesitancy: Clarifying a Theoretical Framework for an Ambiguous Notion. PLoS Curr..

[41] Goldenberg M.J. (2021). Vaccine Hesitancy: Public Trust, Expertise, and the War on Science.

[42] Razai M.S., Chaudhry U.A.R., Doerholt K., Bauld L., Majeed A. (2021). COVID-19 Vaccination Hesitancy. BMJ.

[43] Webb Hooper M., Nápoles A.M., Pérez-Stable E.J. (2021). No Populations Left Behind: Vaccine Hesitancy and Equitable Diffusion of Effective COVID-19 Vaccines. J. Gen. Intern. Med..

[44] Robertson E., Reeve K.S., Niedzwiedz C.L., Moore J., Blake M., Green M., Katikireddi S.V., Benzeval M.J. (2021). Predictors of COVID-19 Vaccine Hesitancy in the UK Household Longitudinal Study. Brain Behav. Immun..

[45] (2021). Mask Usage, Social Distancing, Racial, and Gender Correlates of COVID-19 Vaccine Intentions among Adults in the US. PLoS ONE.

[46] Yoda T., Katsuyama H. (2021). Willingness to Receive COVID-19 Vaccination in Japan. Vaccines.

[47] Akbas Gunes N. (2020). Parents’ Perspectives about Vaccine Hesitancies and Vaccine Rejection, in the West of Turkey. J. Pediatr. Nurs..

[48] Pl R., Ml P., Ml K. (2020). Acceptability of a COVID-19 Vaccine among Adults in the United States: How Many People Would Get Vaccinated?. Vaccine.

[49] Huang C.-L., Chen J.-Y., Lin X.-Q., Deng J.-S., Tung T.-H., Zhu J.-S. (2023). Parents’ Willingness to Pay for Their Children’s COVID-19 Vaccine in Taiwan, China: A Cross-Sectional Study. Hum. Vaccines Immunother..

[50] Shen X., Wu X., Deng Z., Liu X., Zhu Y., Huang Y., Deng Y., Tian Q., Gan Y., Gong Y. (2022). Analysis on Vaccine Hesitation and Its Associated Factors among Parents of Preschool Children in Songgang Street, Shenzhen. Sci. Rep..

[51] Cooper A., Arend U., Eberleh E., Pitschke K. (1999). The Inmates Are Running the Asylum. Software-Ergonomie ’99: Design von Informationswelten.

[52] Brickey J., Walczak S., Burgess T. (2012). Comparing Semi-Automated Clustering Methods for Persona Development. IEEE Trans. Softw. Eng..

[53] User Profiles in Organizational Environments. https://schlr.cnki.net/en/Detail/index/journal.

[54] Quintana R.M., Haley S.R., Levick A., Holman C., Hayward B., Wojan M. (2017). The Persona Party: Using Personas to Design for Learning at Scale. Proceedings of the 2017 CHI Conference Extended Abstracts on Human Factors in Computing Systems.

[55] Tan H., Peng S., Liu J.-X., Zhu C.-P., Zhou F. (2022). Generating Personas for Products on Social Media: A Mixed Method to Analyze Online Users. Int. J. Hum. Comput. Interact..

[56] Xu Y., Lee M.J. (2020). Identifying Personas in Online Shopping Communities. Multimodal Technol. Interact..

[57] Mulder S., Yaar Z. (2006). The User Is Always Right: A Practical Guide to Creating and Using Personas for the Web (VOICES).

[58] An J., Kwak H., Jung S., Salminen J., Jansen B.J. (2018). Customer Segmentation Using Online Platforms: Isolating Behavioral and Demographic Segments for Persona Creation via Aggregated User Data. Soc. Netw. Anal. Min..

[59] Almeshari M., Dowell J., Nyhan J. (2021). Museum Mobile Guide Preferences of Different Visitor Personas. J. Comput. Cult. Herit..

[60] Yoo S., Lee K. (2017). A Data-Driven Approach to Identifying Music Listener Groups Based on Users’ Playrate Distributions of Listening Events. Proceedings of the Adjunct Publication of the 25th Conference on User Modeling, Adaptation and Personalization.

[61] ten Klooster I., Wentzel J., Sieverink F., Linssen G., Wesselink R., van Gemert-Pijnen L. (2022). Personas for Better Targeted EHealth Technologies: User-Centered Design Approach. JMIR Hum. Factors.

[62] Haldane V., Koh J.J.K., Srivastava A., Teo K.W.Q., Tan Y.G., Cheng R.X., Yap Y.C., Ong P.-S., Dam R.M.V., Foo J.M. (2019). User Preferences and Persona Design for an MHealth Intervention to Support Adherence to Cardiovascular Disease Medication in Singapore: A Multi-Method Study. JMIR mHealth uHealth.

[63] Haupt M.R., Weiss S.M., Chiu M., Cuomo R., Chein J.M., Mackey T. (2022). Psychological and Situational Profiles of Social Distance Compliance during COVID-19. J. Commun. Healthc..

[64] Massey P.M., Chiang S.C., Rose M., Murray R.M., Rockett M., Togo E., Klassen A.C., Manganello J.A., Leader A.E. (2021). Development of Personas to Communicate Narrative-Based Information About the HPV Vaccine on Twitter. Front. Digit. Health.

[65] Yin H., Song X., Yang S., Li J. (2022). Sentiment Analysis and Topic Modeling for COVID-19 Vaccine Discussions. World Wide Web.

[66] Mäntylä M.V., Graziotin D., Kuutila M. (2018). The Evolution of Sentiment Analysis—A Review of Research Topics, Venues, and Top Cited Papers. Comput. Sci. Rev..

[67] Tavoschi L., Quattrone F., D’Andrea E., Ducange P., Vabanesi M., Marcelloni F., Lopalco P.L. (2020). Twitter as a Sentinel Tool to Monitor Public Opinion on Vaccination: An Opinion Mining Analysis from September 2016 to August 2017 in Italy. Hum. Vaccine Immunother..

[68] Gao H., Guo D., Wu J., Zhao Q., Li L. (2021). Changes of the Public Attitudes of China to Domestic COVID-19 Vaccination After the Vaccines Were Approved: A Semantic Network and Sentiment Analysis Based on Sina Weibo Texts. Front. Public Health.

[69] Ding J., Wang A., Zhang Q. (2023). Mining the Vaccination Willingness of China Using Social Media Data. Int. J. Med. Inform..

[70] Ansari M.T.J., Khan N.A. (2021). Worldwide COVID-19 Vaccines Sentiment Analysis Through Twitter Content. Electron. J. Gen. Med..

[71] Jang H., Rempel E., Roe I., Adu P., Carenini G., Janjua N.Z. (2022). Tracking Public Attitudes Toward COVID-19 Vaccination on Tweets in Canada: Using Aspect-Based Sentiment Analysis. J. Med. Internet Res..

[72] Monselise M., Chang C.-H., Ferreira G., Yang R., Yang C.C. (2021). Topics and Sentiments of Public Concerns Regarding COVID-19 Vaccines: Social Media Trend Analysis. J. Med. Internet Res..

[73] Yousef M., Dietrich T., Rundle-Thiele S. (2022). Actions Speak Louder Than Words: Sentiment and Topic Analysis of COVID-19 Vaccination on Twitter and Vaccine Uptake. JMIR Form. Res..

[74] Rahmanti A.R., Chien C.-H., Nursetyo A.A., Husnayain A., Wiratama B.S., Fuad A., Yang H.-C., Li Y.-C.J. (2022). Social Media Sentiment Analysis to Monitor the Performance of Vaccination Coverage during the Early Phase of the National COVID-19 Vaccine Rollout. Comput. Methods Programs Biomed..

[75] Sun K., Wang H., Zhang J. (2022). The Impact Factors of Social Media Users’ Forwarding Behavior of COVID-19 Vaccine Topic: Based on Empirical Analysis of Chinese Weibo Users. Front. Public Health.

[76] Zhao J., Wu W., Zhang X., Qiang Y., Liu T., Wu L. (2014). A Short-Term Trend Prediction Model of Topic over Sina Weibo Dataset. J. Comb. Optim..

[77] Song T., Huang J., Tan Y., Yu Y. (2019). Using User- and Marketer-Generated Content for Box Office Revenue Prediction: Differences Between Microblogging and Third-Party Platforms. Inf. Syst. Res..

[78] Shi Z., Rui H., Whinston A.B. (2014). Content Sharing in a Social Broadcasting Environment: Evidence from Twitter. MIS Q. Manag. Inf. Syst..

[79] Liu J., Lu S., Lu C. (2021). Exploring and Monitoring the Reasons for Hesitation with COVID-19 Vaccine Based on Social-Platform Text and Classification Algorithms. Healthcare.

[80] Umar P., Akiti C., Squicciarini A., Rajtmajer S., Dong Y., Kourtellis N., Hammer B., Lozano J.A. (2021). Self-Disclosure on Twitter During the COVID-19 Pandemic: A Network Perspective. Machine Learning and Knowledge Discovery in Databases. Applied Data Science Track.

[81] King G., Zeng L. (2001). Logistic Regression in Rare Events Data. Political Analysis.

[82] Yang X. (2005). A Simulation Study of Logistic Regression and RareEvents Logistic Regression Model. Master’s Thesis.

[83] Salmerón R., García C.B., García J. (2018). Variance Inflation Factor and Condition Number in Multiple Linear Regression. J. Stat. Comput. Simul..

[84] García C.B., García J., López Martín M.M., Salmerón R. (2015). Collinearity: Revisiting the Variance Inflation Factor in Ridge Regression. J. Appl. Stat..

[85] Ahad N.A., Yahaya S.S.S., Yin L.P. (2016). Robustness of S1 Statistic with Hodges-Lehmann for Skewed Distributions. AIP Conf. Proc..

[86] Elliott A.C., Hynan L.S. (2011). A SAS^®^ Macro Implementation of a Multiple Comparison Post Hoc Test for a Kruskal–Wallis Analysis. Comput. Methods Programs Biomed..

[87] Gefen D., Straub D.W. (1997). Gender Differences in the Perception and Use of E-Mail: An Extension to the Technology Acceptance Model. MIS Q..

[88] Cross C.P., Copping L.T., Campbell A. (2011). Sex Differences in Impulsivity: A Meta-Analysis. Psychol. Bull..

[89] Sl K., I M., En F. (2015). Sex-Based Differences in Immune Function and Responses to Vaccination. Trans. R. Soc. Trop. Med. Hyg..

[90] Heidari S., Palmer-Ross A., Goodman T. (2021). A Systematic Review of the Sex and Gender Reporting in COVID-19 Clinical Trials. Vaccines.

[91] McLenon J., Rogers M.A.M. (2019). The Fear of Needles: A Systematic Review and Meta-Analysis. J. Adv. Nurs..

[92] Skjefte M., Ngirbabul M., Akeju O., Escudero D., Hernandez-Diaz S., Wyszynski D.F., Wu J.W. (2021). COVID-19 Vaccine Acceptance among Pregnant Women and Mothers of Young Children: Results of a Survey in 16 Countries. Eur. J. Epidemiol..

[93] Huang J., Chan S.C., Ko S., Wang H.H.X., Yuan J., Xu W., Zheng Z.-J., Xue H., Zhang L., Jiang J.Y. (2022). Factors Associated with Vaccination Intention against the COVID-19 Pandemic: A Global Population-Based Study. Vaccines.

[94] Orenius T., LicPsych, Säilä H., Mikola K., Ristolainen L. (2018). Fear of Injections and Needle Phobia Among Children and Adolescents: An Overview of Psychological, Behavioral, and Contextual Factors. SAGE Open Nurs..

[95] Fu W., Sivajohan B., McClymont E., Albert A., Elwood C., Ogilvie G., Money D. (2022). Systematic Review of the Safety, Immunogenicity, and Effectiveness of COVID-19 Vaccines in Pregnant and Lactating Individuals and Their Infants. Int. J. Gynaecol. Obstet..

[96] Muyldermans J., De Weerdt L., De Brabandere L., Maertens K., Tommelein E. (2022). The Effects of COVID-19 Vaccination on Lactating Women: A Systematic Review of the Literature. Front. Immunol..

[97] Escudero C., Prieto-Montaño P., Audicana M.T. (2022). Adverse Reactions to Anti-Infective Vaccines: An Emerging Problem in the COVID-19 Era. Curr. Treat. Opt. Allergy.

[98] Schinas G., Polyzou E., Mitropetrou F., Pazionis A., Gogos C., Triantos C., Akinosoglou K. (2022). COVID-19 Vaccination in Patients with Chronic Liver Disease. Viruses.

[99] Zhao Y., Du J., Li Z., Xu Z., Wu Y., Duan W., Wang W., Zhang T., Xu J., Wu H. (2023). It Is Time to Improve the Acceptance of COVID-19 Vaccines among People with Chronic Diseases: A Systematic Review and Meta-Analysis. J. Med. Virol..

[100] Moyer-Gusé E., Robinson M.J., Mcknight J. (2018). The Role of Humor in Messaging about the MMR Vaccine. J. Health Commun..

[101] Zhou Z., Zhu Y., Chu M. (2022). Role of COVID-19 Vaccines in SARS-CoV-2 Variants. Front. Immunol..

[102] China|RSF. https://rsf.org/en/country/china.

